# Editorial: Legume intercropping and rotation: impacts on soil microbiomes

**DOI:** 10.3389/fmicb.2026.2006730

**Published:** 2026-10-14

**Authors:** Muhammad Tayyab, Ziqin Pang, Mir Muhammad Nizamani, Yongmei Zhou, Sher Muhammad

**Affiliations:** 1 Shantou University, Shantou, China; 2 Université de Reims Champagne-Ardenne (URCA), Reims, France; 3 Xianghu Laboratory, Hangzhou, China; 4 Government College University, Faisalabad, Pakistan

**Keywords:** crop rotation, legume intercropping, microbial diversity, nitrogen fixation, soil microbiome, sustainable agroecosystems

Soil microbiomes underpin nutrient cycling, organic matter turnover, disease suppression, and plant resilience, and are central to sustainable agroecosystems. Legumes, through symbiotic nitrogen fixation and rhizodeposition, are well positioned to influence soil microbial communities and the services they provide. This Research Topic brings together eight Original Research contributions, six addressing legumes or legume-associated cropping systems and two extending the scope to non-legume medicinal cultivation systems. Two limitations frame the synthesis. First, the Reviews, Mini Reviews, Methods, and Perspectives invited by the Topic are not represented. Second, the evidence is geographically concentrated: seven studies were conducted in China and one in the Peruvian Amazon, and intercropping is represented considerably more strongly than crop rotation.

Legume rotations and cereal–legume intercropping form the largest group of contributions. Nan et al. found that wheat–peanut and wheat–soybean rotations improved wheat yield and quality relative to wheat–maize, with the wheat–peanut system producing the strongest gains, accompanied by enrichment of Acidobacteria and Chloroflexi and greater complexity of fungal co-occurrence networks. In soybean–maize strip intercropping, Liu et al. reported that maize yield was maintained and *nifH* gene abundance increased in the soybean rhizosphere, whereas nitrogen-fixer diversity and richness declined (Shannon-18.2%, Chao1–12.5%). Reducing nitrogen input by 25% increased Shannon diversity by 15.7%, primarily through greater evenness rather than increased richness. This decoupling of abundance from diversity is among the most instructive findings in the collection. In a maize–peanut system, Zheng et al. found that phosphorus fertilization and intercropping increased productivity and were associated with greater relative abundance of *Nitrospirae* and *Mortierellomycota* and reduced relative abundance of Ascomycota, a phylum containing numerous plant-pathogenic taxa; the intercropping yield advantage rose by 60.77%, although the gain in land equivalent ratio was modest (+2.54%). Wu et al. showed that combining chemical and microbial fertilizers in sugarcane–peanut intercropping increased microbial diversity relative to chemical fertilizer alone, enriched rhizobia, increased soil organic

matter, and alleviated acidification. Together, these studies illustrate that microbiome responses to crop diversification are closely intertwined with nutrient management.

Perennial systems are represented by Liu et al., who found that intercropping tea with leguminous green manures, particularly hairy vetch, was associated with greater nutrient availability, altered enzyme activities, and changes in predicted microbial functional potential related to carbon, nitrogen, and phosphorus cycling. Together with the annual-system studies, this suggests that legume-associated shifts in community composition and predicted function can occur across production systems, although much of the evidence remains associative and requires direct functional validation.

Two contributions broaden the scope beyond conventional legume cropping. Zheng et al. compared monoculture *Polygonatum cyrtonema* with intercropping involving *Paris polyphylla* or *Ganoderma lucidum*. Both systems promoted rhizosphere nutrient accumulation, but neither significantly affected *P. cyrtonema* yield; only intercropping with *P. polyphylla* improved selected quality traits, including water-soluble extract (+2.8%) and polysaccharide content (+12.1%). By contrast, Su et al. found that intercropping *P. polyphylla* with *G. lucidum* increased available potassium by 50.28%, strongly enriched *Trichoderma*, and increased yield by 51.30% together with polyphyllin content, whereas intercropping with *P. cyrtonema* enriched nitrogen-fixing taxa and increased available phosphorus by 26.78%. These contrasting responses reinforce that intercropping effects are system-specific rather than universally positive.

Ramos Armaulia et al. broaden the methodological and geographical scope of the collection. From *Inga* sp. nodules, *Quararibea* sp. roots, and rhizosphere soil in cadmium-affected cocoa agroforestry systems in the Peruvian Amazon, 93 bacterial isolates were obtained, of which 53 grew under cadmium screening and 12 yielded high-quality 16S rRNA sequences. No rhizobia sensu stricto were recovered from *Inga* nodules; the recovered nodule isolates were non-rhizobial endophytes. Cadmium tolerance was strongly strain-dependent, with hormetic responses in some isolates. The authors caution that the apparent absence of rhizobia may reflect cultivation conditions rather than true ecological absence, underscoring the value of complementary culture-independent approaches.

Several cross-cutting observations emerge. Legume-based diversification was frequently associated with improved soil nutrient status, shifts toward potentially beneficial microbial taxa, and enhanced crop performance, but responses were strongly context-dependent and varied with cropping system, soil conditions, fertilizer management, companion species, and microbial groups. Microbial abundance, diversity, and predicted function did not necessarily respond in parallel. Reduced nitrogen inputs may, under some conditions, support microbial diversity while reshaping *nifH*-harboring communities. The collection spans culture-dependent isolation, amplicon sequencing, functional-gene quantification, microbial fingerprinting, functional prediction, and enzyme assays. This methodological breadth is a strength, but it also constrains direct quantitative comparison among studies.

Future work should move beyond descriptive community profiling toward stronger functional and causal inference. Priorities include culture-independent verification of rhizobial occurrence in legume nodules under environmental stress; multi-year rotation experiments, which remain scarce relative to intercropping studies; direct validation of microbial functions inferred from sequencing; and explicit linkage of community structure with expressed genes, nutrient fluxes, and crop performance. Coordinated multi-site experiments integrating microbiome analyses with diversified cropping trials under field conditions will be valuable for determining whether these effects are reproducible across soils, climates, and management systems.

Collectively, the studies in this Research Topic demonstrate both the potential and the complexity of managing soil microbiomes through crop diversification. Rather than revealing a single microbial signature of successful intercropping or rotation, they show that plant identity, nutrient management, environmental context, and microbial interactions jointly determine outcomes. We thank all authors, reviewers, and the Frontiers editorial team for their contributions and hope that this collection stimulates further interdisciplinary research linking crop diversification, microbial ecology, and sustainable soil management.

